# A Convenient Fluorogenic Detection Strategy for Phosphorothioate Modification of DNA Through Photocatalytic Oligonucleotide-Templated Reaction

**DOI:** 10.3390/biom15060752

**Published:** 2025-05-23

**Authors:** Nannan Jing, Yantian Qin, Xinli Fan, Qian Wang, Jing Wang, Fuping You, Xinjing Tang

**Affiliations:** 1 School of Basic Medical Sciences, Peking University, No. 38 Xueyuan Rd., Beijing 100191, China; jingnn2015@bjmu.edu.cn (N.J.); fupingyou@bjmu.edu.cn (F.Y.); 2 State Key Laboratory of Natural and Biomimetic Drugs, School of Pharmaceutical Sciences, Peking University, No. 38 Xueyuan Rd., Beijing 100191, Chinawangjing1988@bjmu.edu.cn (J.W.)

**Keywords:** fluorescence probe, phosphorothioate modifications, sequence specificity, oligonucleotide-templated reactions, fluorogenic oligonucleotide probes

## Abstract

DNA phosphorothioate (PT) modifications, characterized by the replacement of a non-bridging phosphate oxygen atom with a sulfur atom, are widely observed in bacterial genomes. Sensitive detection of phosphorothioate is crucial for elucidating their biological roles and functions. Herein, we developed an innovative method that leverages oligonucleotide-templated reactions (OTRs) and fluorogenic oligonucleotide probes. By optimizing temperature, probe sequence length, and the relative distance between PT position and the fluorophore probe, we achieved sensitive detection for DNA PT modifications through fluorogenic signal amplification, which provides an efficient and cost-effective approach for sensitive detection of phosphorothioate-modified DNA.

## 1. Introduction

Deoxyribonucleic acid (DNA) is the fundamental molecule responsible for preserving and conveying genetic information across nearly all known biological organisms [1]. In addition to nucleobases and deoxyribose, the phosphodiester backbone constitutes a unique functional element of DNA [2]. The native DNA backbone linkage is usually the phosphodiester type, which is known for its vulnerability to nuclease activity. However, the phosphorothioate (PT) modification, involving the substitution of a non-bridging phosphate oxygen atom with a sulfur atom, can be artificially engineered through chemical methods to enhance the stability of nucleic acids against nuclease attack [3,4]. Unlike natural nucleic acids, PT-modified nucleic acids, owing to the unique positioning of the modification on the backbone, exhibit enhanced enzymatic stability [5] and are more readily taken up by cells, leading to increased bioavailability [6]. Consequently, this PT modification has found extensive application in the design and development of nucleic acid-based drugs and probes [7].

For a long time, PT-modified nucleic acids have been obtained only through artificial synthesis. The initial detection of natural PT modifications was triggered by the observation of a unique DNA degradation phenotype during electrophoretic analysis of genomic DNA extracted from the microorganism *streptomyces lividans 1326* [8]. This modification was later identified as a sulfur-to-oxygen substitution, a conclusion supported by the detection of isotopes during mass spectrometric analysis [9]. Currently, all physiologically identified PT inter-nucleotide linkages display the unique stereochemistry of the Rp configuration [9,10], which contrasts with the Sp or Rp configurations accessible through artificial synthesis [11]. DNA PT modifications have been detected in a diverse range of bacteria and archaea, yet they have not been observed in eukaryotes as of the current date [12,13,14,15].

The physiological function and evolutionary significance of DNA PT modifications remain largely enigmatic. However, recent studies have begun to elucidate their role in facilitating bacterial adaptation to environmental stresses and have highlighted their potential applications [16,17,18]. Research has shown that DNA PT modifications broaden the range of conditions under which bacteria can grow, allowing them to withstand multiple stressors such as extreme temperatures, high pressure, varying salinity, pH extremes, X-ray exposure, ultraviolet (UV) radiation, and heavy metals [17]. Moreover, this modification exhibits antioxidant properties, shielding genomic DNA and sensitive enzymes from oxidative damage within the cell [18]. The specificity of sequence recognition around modification positions has been examined in some studies [14], revealing that such post-replicative PT modifications of DNA are sequence-selective and stereospecific [13]. The preferred modification positions are often associated with specific consensus sequences, which are targeted by the Dnd protein machinery [16]. PT modifications are also implicated in bacterial defense mechanisms, resembling restriction–modification systems that safeguard bacteria against foreign genetic material [19,20]. Certain restriction enzymes have been shown to specifically recognize PT-modified DNA, binding to it via their DNA sulfur-binding domain (SBD) with high affinity [21]. PT modifications are known to enhance the resistance of DNA to nuclease degradation and are implicated in various physiological processes [7].

The detection and analysis of DNA PT modifications are essential for further elucidating their biological functions [17,18]. Furthermore, the detection of PT modifications of DNA also has significant implications for the development of new antibiotics and therapeutic strategies targeting bacterial infections. Several methods were developed to reveal PT modifications in bacterial genomes. A liquid chromatography–tandem mass spectrometry (LC-MS/MS) technique was established for quantifying PT-linked dinucleotides that were generated from prokaryotic genome hydrolysis with nuclease P1, indicating a diverse range of unique PT sequence contexts and three discrete genomic frequencies in a wide range of bacteria [10]. A selective fluorescent analytical method was also developed to achieve the quantification of total PT contents in whole bacterial DNA and found about 455 PTs per million DNA molecules [22]. Those approaches have been optimized to easily quantify the frequency of DNA PT modifications but are unable to locate the PT position. Subsequently, natural PT modifications have led to the development of methods derived from iodine-induced specific cleavage for identification and quantitation of PT modifications [23,24]. These methods include Iodine-induced Cleavage Assay (ICA), Iodine-induced Cleavage Deep Sequencing (ICDS), and Phosphorothioate Iodine-induced Cleavage Sequencing (PT-IC-Seq). PT-modified sites in the genome could be mapped using Single-Molecule Real-Time (SMRT) sequencing [25] and deep sequencing combined with iodine-induced cleavage methods [26,27]. Nevertheless, these methods also have shortcomings, such as the relatively high cost, relative quantification of specific PT-modified positions in samples, etc.

**Figure 1:**
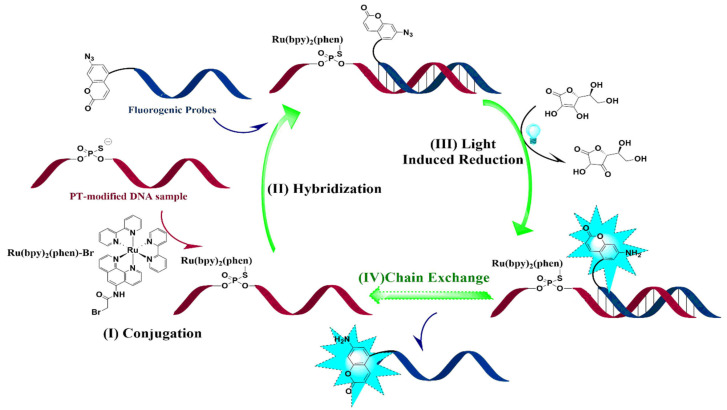
Schematic diagram of the combination of fluorogenic nucleic acid probes and OTRs in the quantification of PT modification frequency. (**I**) Conjugation: DNA sample was treated with Ru(bpy)_2_(phen)-Br. (**II**) Hybridization: DNA sample was treated with fluorogenic nucleic acid probes. (**III**) Light-induced reduction: The reduction of 7-azido-coumarin was quantified by fluorescence detection. (**IV**) Chain exchange: Start with new fluorogenic cycles.

DNA probe technology is a widely used DNA sequence detection technique in molecular biology research, which relies on the principle of complementary base pairing to achieve highly accurate recognition between DNA molecules. This technique exhibits high sequence specificity, and DNA probes are easy to synthesize and modify, thus holding good application prospects. The discovery of various chemical reactions using oligonucleotides as templates has provided a new theoretical basis for the design of DNA probes. Many probes based on DNA-templated reactions have been successfully applied in gene detection. Among them, probes that rely on non-linked mode reactions can catalyze hundreds or thousands of reactions through template chains, thereby achieving signal amplification without the need for sequence amplification techniques such as PCR. These probes have received widespread attention from researchers. In order to efficiently and cost-effectively identify PT-modified positions in specific sequences, we developed a novel strategy through the combination of chain exchange and photocatalytic oligonucleotide-templated reactions (Figure 1) inspired by the reported oligonucleotide template reaction using ruthenium (II) complexes to reduce 7-azido-coumarin. The ruthenium (II) photocatalyst can be ligated to a template DNA strand through phosphorothiolation, which can then elicit the light-mediated reduction of a fluorogenic-modified antisense strand, measured by its consequent fluorescence. This convenient and economical approach is promising to detect sequence-specific PT-modified positions, which enhances the sensitivity through a signal amplification mechanism to meet the requirements for detecting low-frequency PT-modified positions.

## 2. Materials and Methods

### 2.1. Materials

The DNA oligonucleotides in Table 1 were purchased from Yaoyuan Biotechnology (Shanghai, China). 7-Aminocoumarin-4-acetic acid was purchased from Bide Pharmatech (Shanghai, China). cis-Dichlorobis (2,2′-bipyridine) ruthenium (II) dihydrate was purchased from Meryer Chemical Technology (Hong Kong, China).

### 2.2. The Modified Method of PT-Modified DNA Sample (Figure S3a)

Bromoacetyl bromide was couple to the amino moiety of 5-amino-1, 10-phenanthroline (Accela ChemBio, Shanghai, China) to obtain Ru(bpy)_2_(phen)-Br (Figure S1). The PT-modified DNA samples (GpsA-1, GpsA-2, and GpsA-3) were prepared using the following method: 1 mg of Ru(bpy)_2_(phen)-Br was dissolved in 100 μL of acetonitrile to prepare stock solution (10 mM). Subsequently, 20 nmol of the PT-modified DNA samples was dissolved in 100 μL PBS buffer (10 mM, pH = 8.0). The two solutions were then mixed thoroughly and allowed to react in the dark at room temperature for 2 h. The solutions were first filtrated with 3 kDa cutoff using DEPC water containing 20% acetonitrile as the eluent. All residues were purified using a Waters high-performance liquid chromatography (HPLC) system under a reversed-phase column (C18) and further characterized using electrospray ionization mass spectrometry (ESI-MS).

### 2.3. Synthesis of Fluorogenic Nucleic Acid Probes (Figure S3b)

The probe sequences 8nt-NH_2_, 11nt-NH_2_, 15nt-NH_2_, and MM-NH_2_, each bearing amino groups at the 3′ end, were conjugated with 7-azido-coumarin activated NHS ester (7-AzC) via the following procedure: Initially, 1 mg of 7-azido-coumarin active ester in 100 μL of DMF was mixed with 30 nmol of the above oligonucleotides (100 mM PBS buffer, pH = 7.2–7.4) at room temperature in the dark for 6 h. Then, the solution was dialyzed with a molecular weight cutoff of 1000 Da. All products (8nt-cou, 11nt-cou, 15nt-cou and MM-cou) were further purified using HPLC and characterized using ESI-MS.

### 2.4. The Method of Fluorescence Detection

The templated oligonucleotides (GpsA-Ru-1, GpsA-Ru-2, and GpsA-Ru-3) and the probe oligonucleotides (8nt-cou, 11nt-cou, 15nt-cou and MM-cou) were each individually dissolved in PBS buffer (100 mM, pH = 7.2–7.4) as 100 μM stock solution and were then diluted to the different concentrations with buffer containing 15 mM ascorbic acid and 0.05% Tween 20 according to detection conditions. Subsequently, the GpsA-Ru-1, GpsA-Ru-2, or GpsA-Ru-3 solution was added to a black fluorescent enzyme-linked immunosorbent assay plate, followed by the addition of the probe oligonucleotides. The plate was placed on a microplate shaker under irradiation from a blue LED flashlight positioned 30 cm away. The plate was incubated at a temperature of 25 °C or 35 °C while the fluorescence intensity from each well was recorded at intervals of 10 min, 20 min, 30 min, 50 min, 70 min, 100 min, and 120 min. The excitation wavelength was set to 360 nm with an emission wavelength fixed at 460 nm.

## 3. Results and Discussion

### 3.1. Rational Design of Fluorogenic Nucleic Acid Probes in PT Modification Quantification

Genomic mapping of PT modifications indicates that only certain short consensus sequences undergo modification, but the detailed information regarding PT modifications at specific positions is still pending elucidation. According to the previous report of azido moiety reduction catalyzed by the Ru (II) complex [28], we developed a novel strategy through the combination of fluorogenic nucleic acid probes and oligonucleotide-templated reactions (OTRs) to detect the PT modification of nucleic acids (Figure 1): (1) The thiol moiety of PT-modified oligonucleotide reacted with Ru(bpy)_2_(phen)-Br to form an S-C bond by replacing Br, and Ru(bpy)_2_(phen) conjugated oligonucleotides were then obtained. (2) Fluorogenic oligonucleotide probes were hybridized with Ru(bpy)_2_(phen) modified oligonucleotide and 7-azido-coumarin (7-AzC) in these probes that were subsequently reduced to 7-amino-coumarin, resulting in fluorescence emission. The new fluorogenic nucleic acid probes in solution exchanged with the 7-AzC oligonucleotide product to induce new fluorogenic events, thereby achieving fluorescence signal amplification for sensitive detection.

### 3.2. Optimization of Relative Distance Between PT-Modified Position and 3′ End of Probe Sequence

A previously reported PT-modified oligonucleotide sequence previously found in *Escherichia coli* B7a was selected as the detection target (GpsA-0), where the position of PT modification was located between nucleosides G and A. Thus, we first optimized the distance between the PT-modified position and the 3′ end 7-AzC of the fluorogenic probe sequences due to the fact that the distance would greatly affect the oligonucleotide template reaction of 7-AzC reduction catalyzed by Ru(bpy)_2_(phen). By repositioning the GAGA sequence to the 5′ end of GpsA-0 with PT modification (GpsA-template), three oligodeoxynucleotide templates were obtained with Ru (II) conjugation: GpsA-Ru-1, GpsA-Ru-2, and GpsA-Ru-3. Upon pairing with the fluorogenic oligonucleotide probe (8nt-cou), the PT-modified positions of these three oligodeoxynucleotide templates were separated from the 3′ end of the 8nt-cou probe by zero, two, and four nucleotides, respectively.

**Figure 2:**
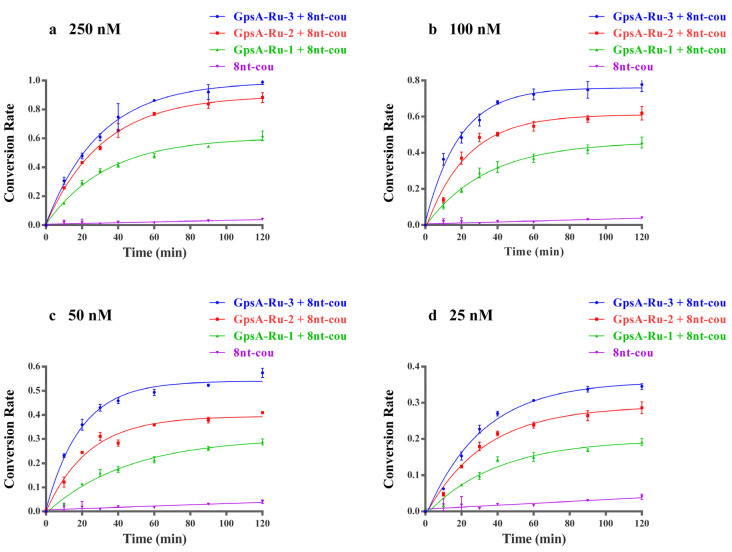
First-order dynamics curves of three oligodeoxynucleotides with different PT modification sites (GpsA-Ru-1, GpsA-Ru-2, GpsA-Ru-3) detected by 8nt-cou (500 nM) at concentrations of 250 nM (**a**), 100 nM (**b**), 50 nM (**c**), and 25 nM (**d**) (λex = 360 nm, λem = 460 nm, T = 25 °C), respectively.

The fluorogenic events of the 8nt-cou probe with three PT-modified oligodeoxynucleotide templates were investigated at concentrations from 25 nM to 250 nM. The fluorescence intensity of the above samples was monitored during the reaction process with the treatment of a blue LED flashlight. As shown in Figure 2, by plotting the curves of the first-order dynamic model of the reaction, we demonstrated that 8nt-cou had the highest reaction rate constant when detecting template GpsA-Ru-3, and the conversion rate of 7-azido-coumarin with the GpsA-Ru-3 system was also the highest when the reaction reached equilibrium. These results showed that 7-azido-coumarin could approach the Ru (II) complex in the most suitable orientation when the probe 3′ end of 7-azido-coumarin was separated from the PT-modified position without extra nucleotides. With the increase in extra nucleotides, the 7-azido-coumarin and Ru(bpy)_2_(phen) were far away from each other. The collision probability between two functional groups decreased, resulting in a gradual decline in the first-order conversion rate constant (kGpsA-Ru-3 = 0.043 min^−1^ > kGpsA-Ru-2 = 0.037 min^−1^ > kGpsA-Ru-1 = 0.027 min^−1^, as shown in Figure S4). Therefore, we used the probes without extra nucleotides between the 3′ end of the fluorogenic 7-azido-coumarin moiety and the PT-modified position for further evaluation in subsequent experiments.

In addition to the effect of different PT modification positions, the conversion rate was also closely related to the concentration of GpsA-Ru-3, GpsA-Ru-2 and GpsA-Ru-1. As shown in Figure 2, with higher concentrations of GpsA-Ru, higher conversion rates were observed if the 8nt-cou concentration was fixed at 500 nM. To confirm the relationship between the concentration of target oligonucleotide GpsA-Ru and fluorogenic signal intensity, we compared the fluorescence readings obtained by incubating varying concentrations of GpsA-Ru-3 with the probe 8nt-cou under the same experimental conditions. To ensure the full conversion of 7-AzC to fluorescent 7-amino-coumarin, we first incubated the target GpsA-Ru-3 at a concentration of 1 μM with the probe 8nt-cou at a concentration of 500 nM for 2 h, and the maximum fluorescence intensity was obtained. The fluorescence intensity was then measured after incubating 8nt-cou with different concentrations of GpsA-Ru-3 under the same reaction conditions.

**Figure 3:**
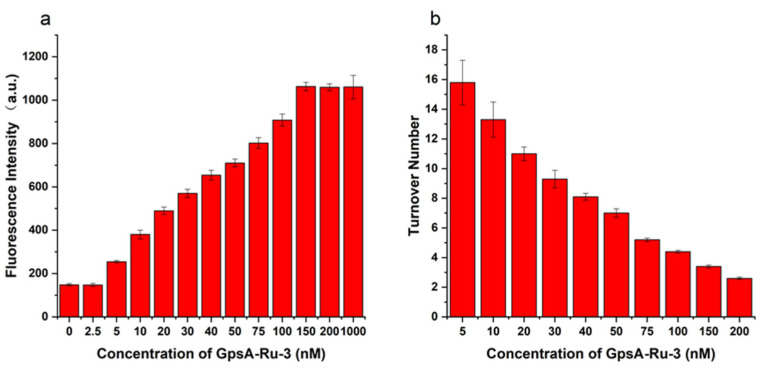
Fluorescence intensity measured after incubation of 8nt-cou with GPSA-Ru-3 sequence with different concentrations for 2 h (**a**), and reaction conversion number of the probe after incubation with GPA-3 sequence at different concentrations for 2 h (**b**) (λex = 360 nm, λem = 460 nm, T = 25 °C).

As shown in Figure 3a, when the GpsA-Ru-3 concentration was 2.5 nM, the fluorescence intensity was almost equal to the background fluorescence intensity within 2 h. After increasing the GpsA-Ru-3 to 5 nM, an obvious detectable fluorescence signal was observed and the fluorescence intensity increased gradually with the higher GpsA-Ru-3 concentration. And the fluorescent signal reached a plateau at a concentration of 150 nM within 2 h, which is the same signal intensity as when GpsA-Ru-3 was overdosed at 1000 nM. This observation indicated that one GpsA-Ru-3 could catalyze the reduction of multiple 8nt-cou oligonucleotide probes. Thus, the turnover numbers for GpsA-Ru-3 at different concentrations were calculated under the same conditions, as shown in Figure 3b. The turnover number was the average number of times that each GpsA-Ru-3 could catalyze the reduction of 7-azido-coumarin of the 8nt-cou probe within 2 h. When the GpsA-Ru-3 concentration was 200 nM, the turnover number was 2.5, indicating the full conversion of 7-AzC on the 8nt-cou probe (500 nM), while when the GpsA-Ru-3 concentration was 5 nM, the turnover number was up to 16 in 2 h, which meant that a longer reaction time might be needed to fully convert 7-azido-coumarin of the 8nt-cou probe to the 7-amino-coumarin moiety. Taken together, this probe design can possibly achieve signal amplification by mediating multiple turnovers of the reduction of the 7-azido-coumarin moiety on the 8nt-cou probe.

### 3.3. Effect of Probe Length on PT Detection

**Figure 4:**
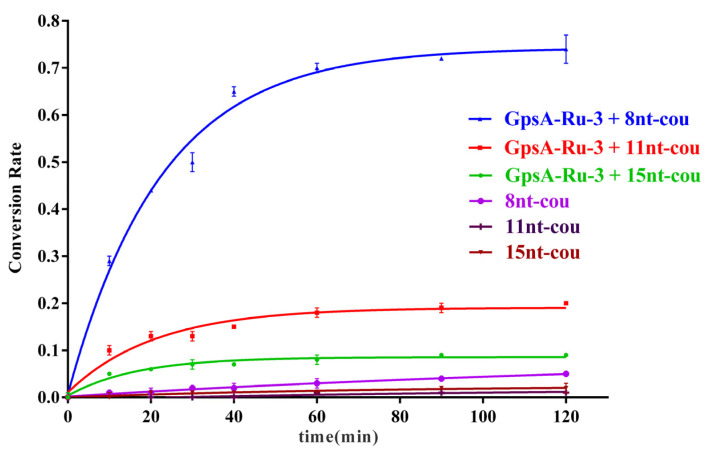
Fluorescence curves of GpsA-Ru-3 (100 nM) detected by 8nt-cou, 11nt-cou, and 15nt-cou (500 nM) over time (λex = 360 nm, λem = 460 nm, T = 25 °C), respectively.

To optimize the effect of the probe oligonucleotide chain length on the detection efficiency of PT modification on oligonucleotides, we further synthesized three 7-azido-coumarin modified oligonucleotide probes of varying lengths (8nt-cou, 11nt-cou, and 15nt-cou) targeting the same sequence (GpsA-Ru-3) under the same experimental conditions. The first-order dynamic model curves were plotted for these three probes (8nt-cou, 11nt-cou, and 15nt-cou, 500 nM) in relation to GpsA-Ru-3 (100 nM) and their detection performance was compared. As illustrated in Figure 4, we observed that the reaction rate constants for these three systems followed the order k15nt-cou (0.061 min^−1^) > k11nt-cou (0.047 min^−1^) > k8nt-cou (0.043 min^−1^). This trend could be attributed to the binding affinity relationships among the three probe sequences with GpsA-Ru-3 (15 nt > 11 nt > 8 nt). A longer oligonucleotide sequence is supposed to more tightly bind the PT-modified nucleic acid, and the Ru(bpy)_2_(phen)-induced azido reduction should be more efficient (15 nt > 11 nt > 8 nt). However, the conversion rate of the 7-AzC moiety significantly decreased once the probe sequence shifted from 8 nt (0.72) to 11 nt (0.19) and 15 nt (0.09), as shown in Figure 3. This phenomenon was ascribed to the lower binding affinity of 8nt-cou with GpsA-Ru-3, which enabled the quick exchange of 8nt-cou and its fluorogenic product and triggered the fluorescence signal amplification with higher turnover numbers.

### 3.4. Effect of Temperature on Detection Performance

**Figure 5:**
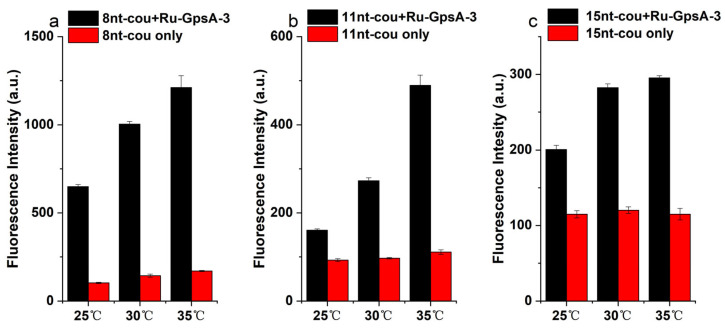
Fluorescence curves of GpsA-Ru-3 (100 nM) detected by (**a**) 8nt-cou, (**b**) 11nt-cou, and (**c**) 15nt-cou (500 nM) at three different temperatures after 2 h, respectively (λex = 360 nm, λem = 460 nm).

The hybridization affinity of GpsA-Ru-3 with oligonucleotide probes (8nt-cou, 11nt-cou, and 15nt-cou) was also affected by the temperature in addition to the complementary sequence length. The temperature dependence of the fluorogenic response for all three probes was investigated. As shown in Figure 5, a gradual increase in the fluorescence signal of the GpsA-Ru-3/8nt-cou solution was observed with temperatures from 25 to 30 to 35 °C, and up to 6.4-fold fluorogenic signal enhancement was achieved at 35 °C, while negligible signal variation was observed for only 8nt-cou under the same condition. The temperature effect was more obvious when 11nt-cou was applied for the detection of GpsA-Ru-3. Around 3.7-fold enhancement in the signal of GpsA-Ru-3/11nt-cou was observed at 35 °C in comparison to a 1.5-fold increase in the above solution at 25 °C. With the longer oligonucleotide probe 15nt-cou, temperature elevation only slightly enhanced the fluorogenic signals of 15nt-cou from 25 to 30 and 35 °C, which indicated that the binding affinity of GpsA-Ru-3/15nt-cou was much higher than that of GpsA-Ru-3/8nt-cou and GpsA-Ru-3/11nt-cou, and 35 °C was not high enough for quick chain exchange. All these data indicated that the turnover numbers of GpsA-Ru-3-induced conversion of the 7-azido-coumarin moiety on oligonucleotide probes was the key factor for the sensitive detection of PT modification of nucleic acid, and the temperature has a large effect on the turnover number of these fluorogenic conversions. The increase in the length of DNA probes is beneficial for the photocatalytic reaction rate but not conducive to the turnover numbers. As shown in Figure 4, the conversion rate has a greater impact; 8nt-cou is the best probe for this template. For other template, we need to balance the stronger binding affinity of longer probe sequences with the higher chain exchange rate of shorter sequences.

### 3.5. Sequence Selectivity of PT Detection Using 8nt-cou

**Figure 6:**
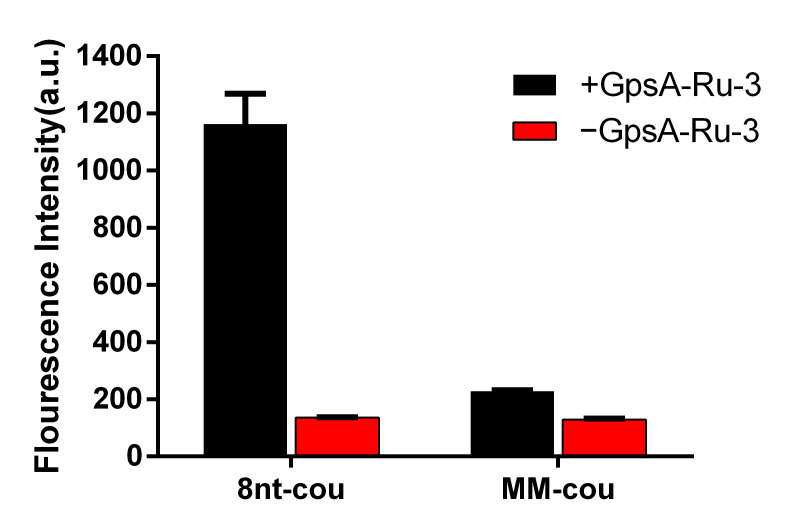
Fluorescence intensities of 8nt-cou and MM-cou were measured after incubation with GPSA-Ru-3 for 2 h (λex = 360 nm, λem = 460 nm, T = 35 °C).

To further investigate the specificity of the target oligonucleotide sequence, we also synthesized a mutated 8nt-cou (MM-cou, Table 1) with a single mismatched nucleotide A to T. The detection performance of both MM-cou and 8nt-cou probes (500 nM) was evaluated for the target sequence GpsA-Ru-3 (100 nM) at 35 °C. As shown in Figure 6, both MM-cou and 8nt-cou without the presence of GpsA-Ru-3 had weak background signals. Upon the addition of GpsA-Ru-3, only slight fluorescence signal enhancement was observed with the MM-cou probe containing a single mismatched nucleotide. As expected, a significant fluorogenic signal from 8nt-cou/GpsA-Ru-3 was achieved, up to 3.5-fold stronger than that of the single mutated MM-cou probe, indicating good sequence specificity. This result confirmed that the detection probe could not only identify the PT modification positions in nucleic acids but also achieve this in a sequence-specific manner.

## 4. Conclusions

Based on the oligonucleotide template reaction catalyzed by the Ru (II) complex, we developed a sensor system for PT modification of nucleic acids, which contained Ru(bpy)_2_(phen) labeling of PT and a fluorogenic reaction with 7-azido-coumarin modified oligonucleotide probes. PT containing oligonucleotides first reacted with Ru(bpy)_2_(phen)-Br via S-C bond formation through SN2 substitution reaction. Then, 7-azido-coumarin modified oligonucleotide probes were added to the above detection solution. The fluorogenic signals from the 7-amino-coumarin moiety could be applied to sense the PT modification through the photocatalytic reduction of an azido moiety using Ru(bpy)_2_(phen). Through optimization of the target/probe oligonucleotide sequences, a sensitive detection strategy for DNA sulfur substitution positions was achieved through fluorogenic signal amplification. The effect of the relative distance between the PT position and the probe fluorophore 7-azido-coumarin on the detection performance was explored to confirm the proximity reaction of Ru(bpy)_2_(phen) and 7-azido-coumarin moieties. In addition, among three oligonucleotide probes (8nt-cou, 11nt-cou, and 15nt-cou), the shorter probe, 8nt-cou, showed the best performance in terms of fluorogenic signal amplification at temperatures from 25 to 35 °C, although the longer oligonucleotide probes preferred high temperatures for PT detection. This signal amplification was ascribed to the higher turnover number for the shorter oligonucleotide probe 8nt-cou due to the weaker binding affinity and quick chain exchange of GpsA-Ru-3 and 8nt-cou. Further sequence specificity study confirmed the significant difference in fluorogenic signals for PT detection, even with the single mismatched oligonucleotide probe MM-cou, in comparison to 8nt-cou. With this new OTR sensing system, it is possible to develop a general, efficient and cost-effective approach for detecting PT modification on nucleic acids.

## Figures and Tables

**Table 1:** List of modified DNA sequences and probes synthesized in this study.

Name	Sequence (5′–3′)
GpsA-0	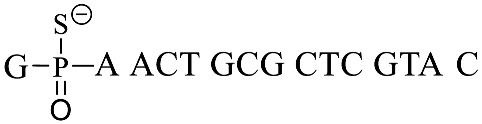
GpsA-template	GAG AGA ACT GCG CTC GTA C
GpsA-Ru-1	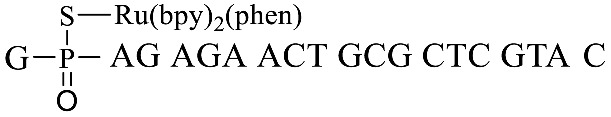
GpsA-Ru-2	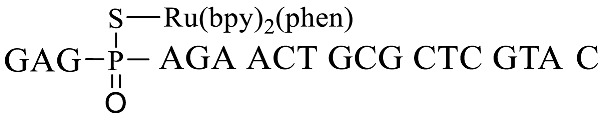
GpsA-Ru-3	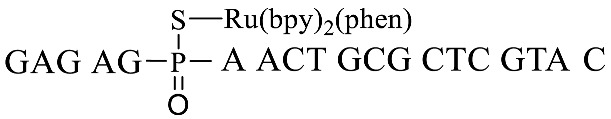
8nt-cou	GCG CAG TT-(7-AzC)
11nt-cou	CGA GCG CAG TT-(7-AzC)
15nt-cou	CGA GCG CAG TT-(7-AzC)
MM-cou	GCG CTG TT-(7-AzC)

## Data Availability

The original contributions presented in this study are included in the article/Supplementary Material. Further inquiries can be directed to the corresponding author.

## Supplementary Materials

The following supporting information can be downloaded at https://www.mdpi.com/article/10.3390/biom15060752/s1: Figure S1: Synthesis method of Ru(bpy)_2_(phen)-Br. Figure S2: Synthesis method of 7-azido-coumarin active ester. Figure S3: (a) Schematic diagram of the sulfur in the PT-modified DNA sample, which was modified by Ru(bpy)_2_(phen). (b) Schematic diagram of the amino-modified 3′ end of probe sequences to attach 7-AzC. Figure S4: Conversion rate. Figures S5–S16: ^1^H NMR, ^13^C NMR or ESI-MS. Table S1. List of modified DNA sequences in this study.
