# Supplementary material for: A Convenient Fluorogenic Detection Strategy for Phosphorothioate Modification of DNA Through Photocatalytic Oligonucleotide-Templated Reaction
Source: Biomolecules. 2025 May 23;15(6):752. doi: 10.3390/biom15060752 (PMC12191357; doi:10.3390/biom15060752)
Supplement: Supplementary file 1 [file biomolecules-15-00752-s001.zip › biomolecules-3470471-supplementary.pdf]

Supporting Information

A Convenient Fluorogenic Detection Strategy for Phos-phorothioate Modification of DNA Through Photocatalyst Ol-igonucleotide-Templated Reaction

Nannan Jing†[1], Yantian Qin†[2], Xinli Fan[2], Qian Wang[2], Jing Wang[2], Fuping You[1], Xinjing Tang*[2]

[1]School of Basic Medical Sciences, Peking University, No. 38 Xueyuan Rd., Beijing 100191, China

[2]State Key Laboratory of Natural and Biomimetic Drugs, Chemical Biology Center and School of Pharmaceutical Sciences, Peking University, No. 38 Xueyuan Rd., Beijing 100191, China

† These authors contributed equally to this work.

* Corresponding Author: xinjingt@bjmu.edu.cn(X.T.); fupingyou@bjmu.edu.cn(F.Y.); wangjing1988@bjmu.edu.cn(J.W.)

1. Synthesis of Ru(bpy)$_2$(phen)-Br

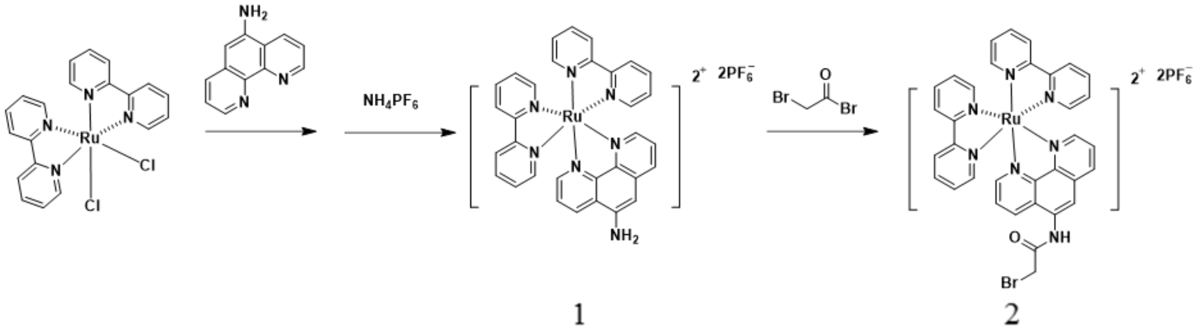

Figure S1. Synthesis method of Ru(bpy)$_2$(phen)-Br

*Synthesis of compound **1***

Cis-dichlorobis(2,2'-bipyridine)ruthenium(II) dihydrate (0.350 g, 0.725 mmol) and 5-amino-1,10-phenanthroline (0.142 g, 0.725 mmol) were added into methanol (25mL), and refluxed under nitrogen for 3 hours. Upon cooling the reaction mixture, the solution was subjected to filtration. Subsequently, a saturated aqueous solution of ammonium hexafluorophosphate was carefully introduced into the filtrate in a dropwise manner until precipitation occurs. The precipitate was collected by filtration, washed

multiple times with ice water and ice ether to obtain a red solid (0.386g, 59%). It was used for the next reaction without further purification. ^{1}H NMR (400 MHz, DMSO-d$_6$) δ 8.92 (d, J = 8.5 Hz, 1H), 8.89 – 8.79 (m, 4H), 8.32 (d, J = 8.1 Hz, 1H), 8.20 (td, J = 8.0, 3.4 Hz, 2H), 8.12 (t, J = 7.9 Hz, 2H), 8.06 (d, J = 5.2 Hz, 1H), 7.87 – 7.78 (m, 3H), 7.65 – 7.51 (m, 6H), 7.39 (dt, J = 9.6, 6.5 Hz, 2H), 7.09 (s, 1H), 6.94 (s, 2H)； ^{13}C NMR (101 MHz, DMSO-d$_6$) δ 157.27, 157.07, 157.00, 152.23, 151.91, 151.83, 151.58, 148.12, 145.78, 140.95, 138.25, 138.25, 134.73, 133.77, 133.06, 128.25, 126.45, 125.29, 124.75, 123.79. MS(m/z): Calcd for $C_{32}H_{25}N_7Ru^{2+}$, 304.33; found, 304.46.

*Synthesis of compound **2***

Under nitrogen protection, compound **1** (75.0 mg, 0.0825 mmol) was dissolved in anhydrous acetonitrile (3.0 mL) and cooled in an ice water bath. Bromoacetyl bromide (11.0 μL, 0.127 mmol) and triethylamine (11.0 μL, 0.165 mmol) was individually dissolved in anhydrous acetonitrile (1 mL) and slowly added them to the above reaction system. The mixture was stirred overnight at room temperature in the dark. The organic solvent was removed via rotary evaporation. The resultant dark red solid was subsequently dissolved in the minimal amount of methanol necessary, followed by filtration to eliminate insoluble materials. Subsequently, a saturated aqueous solution of ammonium hexafluorophosphate was cautiously added dropwise to the filtrate until precipitation occurs, which was then filtered again to collect the precipitate. This precipitate was thoroughly washed several times with ice-cold water, followed by ice-cold ether, and finally vacuum-dried to yield the desired orange-red solid (64.6 mg, 77%). ^{1}H NMR (400 MHz, DMSO-d$_6$) δ 10.87 (s, 1H), 8.9 – 8.73 (m, 6H), 8.65 (s, 1H), 8.30 – 8.02 (m, 6H), 7.95 (t, J = 7.1 Hz, 1H), 7.84 (q, J = 7.0 Hz, 3H), 7.58 (m, 4H), 7.43–7.26 (m, 2H), 4.34 (s, 2H). MS(m/z): Calcd. for $C_{34}H_{26}BrN_7ORu^{2+}$ 364.52; found, 364.27.

2. Synthesis of 7-azido-coumarin (7-AzC) active ester

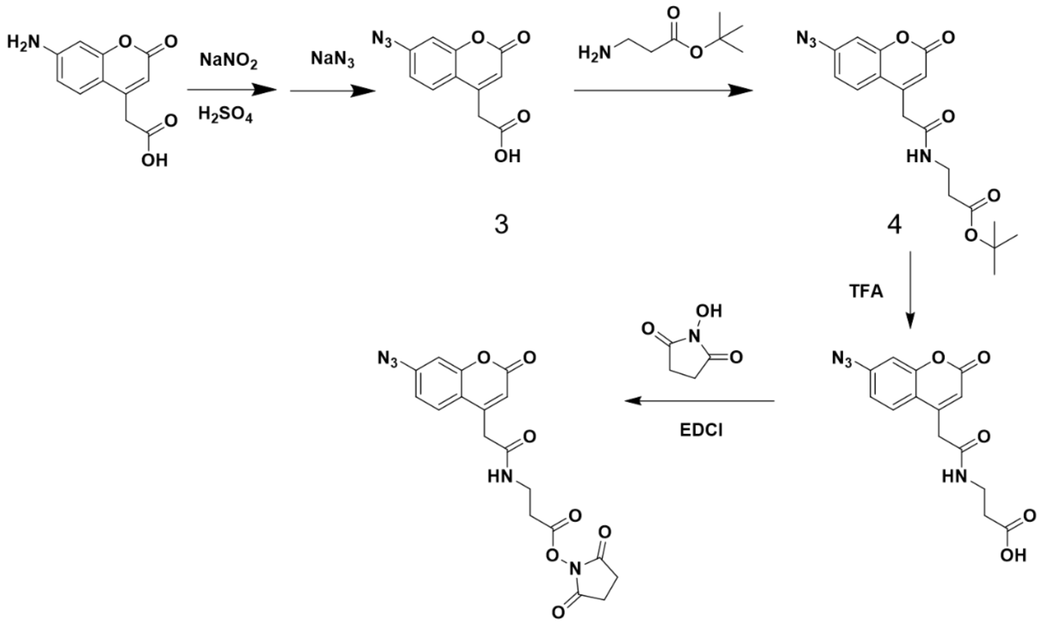

6 5

Figure S2. Synthesis method of 7-azido-coumarin active ester

*Synthesis of compound **3***

7-Aminocoumarin-4-acetic acid (90.0 mg, 0.41 mmol) was suspended in 2 mL of deionized water and cooled in an ice-water bath. Subsequently, 0.5 mL of concentrated sulfuric acid was slowly added. Sodium nitrite ($NaNO_2$, 34.0 mg, 0.48 mmol) was dissolved in 0.6 mL of deionized water and this solution was then slowly dripped into the aforementioned reaction mixture. The mixture was stirred for one hour. Sodium azide (44.0 mg, 0.67 mmol) was added in multiple portions, and the reaction was allowed to proceed overnight at room temperature. Upon completion of the reaction, the mixture was filtered to collect the precipitate, which was washed multiple times with ice-cold water and then vacuum-dried to yield a light-yellow solid (**3**, 75.3 mg, 75%). This solid was used to the next reaction step without further purification. ^{1}H NMR (400 MHz, DMSO-d_6) δ 7.74 (d, J = 8.6 Hz, 1H), 7.21 (d, J = 2.2 Hz, 1H), 7.15 (dd, J = 8.6, 2.2 Hz, 1H), 6.45 (s, 1H), 3.92 (s, 2H); ^{13}C NMR (101 MHz, DMSO-d6) δ 170.99, 159.97, 154.68, 150.14, 143.87, 127.66, 116.60, 116.17, 115.53, 107.46, 40.63, 40.42, 40.21, 40.00, 39.58, 39.38. MS(m/z): Calcd. for $C_{11}H_6N_3O_4$ 245.19; found, 246.03 $[M+H]^+$.

*Synthesis of compound **4***

Compound **3** (245.0 mg, 1.0 mmol) was dissolved in 30 mL of dry tetrahydrofuran. Subsequently, alanine tert-butyl ester hydrochloride (272.0 mg, 1.5 mmol), EDCI (383.0 mg, 2.0 mmol), and TEA (0.40 mL, 3 mmol) were sequentially added to the reaction system. The mixture was stirred overnight at room temperature. After the reaction was complete, the solvent was removed by rotary evaporation. The resultant mixture was analyzed and purified via silica gel column chromatography using a dichloromethane: methanol (v/v, 20:1) eluent. The final product **4** was obtained as a white solid (301.0 mg, 81%).^{1}H NMR (400 MHz, DMSO-d_6) δ 8.33 (t, 1H), 7.78 (d, J = 8.6 Hz, 1H), 7.19 (d, J = 2.2 Hz, 1H), 7.13 (dd, J = 8.6, 2.2 Hz, 1H), 6.37 (s, 1H), 3.70 (s, 2H), 3.26 (q, J = 6.4 Hz, 2H), 2.37 (t, J = 6.4 Hz, 2H), 1.38(s, 9H). MS(m/z): Calcd. for $C_{18}H_{20}N_4O_3$ 372.39; found, 373.15 $[M+H]^+$.

*Synthesis of compound **5***

Compound **4** (100.0 mg, 0.27 mmol) was dissolved in 2 mL of dichloromethane, followed by the addition of 1 mL of trifluoroacetic acid. The mixture was stirred at room temperature overnight. Upon completion of the reaction, ether was introduced to the reaction solution, inducing precipitation. The precipitate was filtered, collected, and washed multiple times with ether. After vacuum drying, a white solid (75.1 mg, 88%) was obtained. Without additional purification, the product **5** was advanced to the subsequent reaction step. ^{1}H NMR (400 MHz, DMSO-d_6) δ 12.27 (s, 1H), 8.36 (t, 1H), 7.76 (d, J = 8.6 Hz, 1H), 7.19 (d, J = 2.3 Hz, 1H), 7.13 (dd, J = 8.5, 2.3 Hz, 1H), 6.38 (s, 1H), 3.71 (s, 2H), 3.27 (q, J = 6.4 Hz, 2H), 2.39 (t, J = 6.4 Hz, 2H). MS(m/z): Calcd. for $C_{14}H_{12}N_4O_5$ 316.08; found, 315.07 $[M-H]^-$.

*Synthesis of compound **6***

Compound **5** (82 mg, 0.26 mmol) was suspended in 30 mL of dry tetrahydrofuran under nitrogen protection and heated to 50 °C until the solid was completely dissolved. N-hydroxy succinimide (34.5 mg, 0.30 mmol) and EDCI (76.7 mg, 0.40 mmol) were dissolved in 5 mL of dry tetrahydrofuran and sequentially added to the reaction system. The mixture was stirred for 4 hours until the reaction was complete. After cooling, the organic solvent was removed by rotary evaporation, and the product was purified by silica gel column chromatography using dichloromethane: ethyl acetate (v/v, 1:1) as the eluent. The final product **6** was obtained as a white solid (58.0 mg, 54%).^{1}H NMR (400 MHz, DMSO-d_6) δ 8.50 (t, 1H), 7.74 (d, J = 8.6 Hz, 1H), 7.20 (d, J = 2.3 Hz, 1H), 7.13 (dd, J = 8.6, 2.3 Hz, 1H), 6.39 (s, 1H), 3.73 (s, 2H), 3.39 (q, J = 6.3 Hz, 2H), 2.88 (t, J = 6.3 Hz, 2H), 2.83 (s, 4H). MS(m/z): Calcd. for $C_{18}H_{14}N_5O_7$ 413.35; found, 414.03 $[M+H]^+$, 436.04 $[M+Na]^+$.

3. Synthesis of PT-modified oligonucleotides and fluorogenic oligonucleotide probes

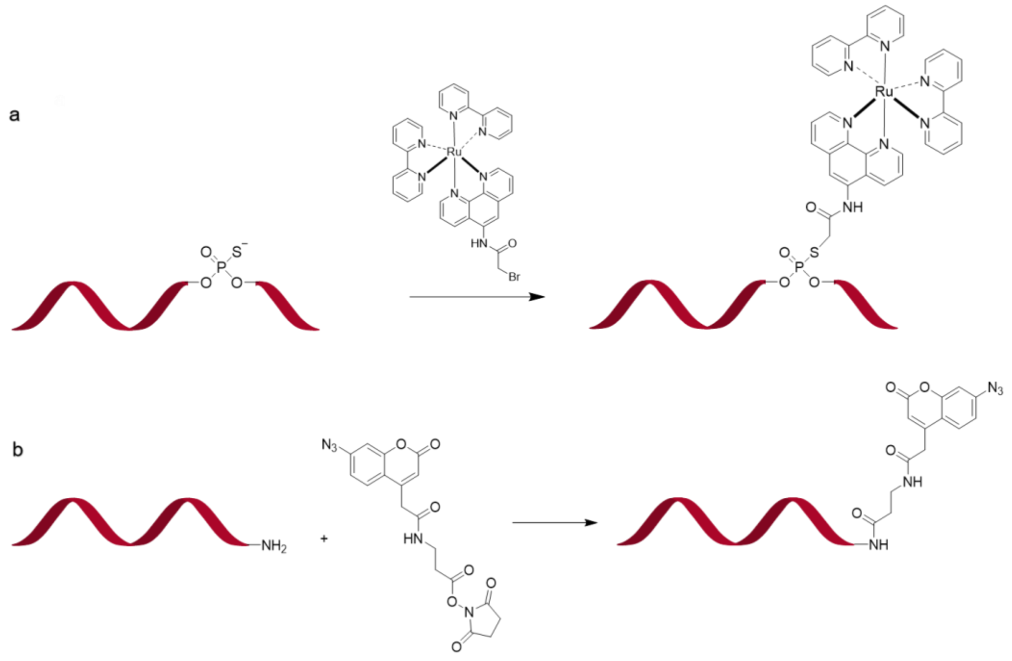

Figure S3 (a)Schematic diagram of substitution of PT-modified oligonucleotides and Ru(bpy)$_2$(phen)Br. (b) Schematic diagram of conjugation of amino modified oligonucleotides probes and compound **6**

(a) Modification of PT-modified oligonucleotides.

The PT-modified DNA samples (GpsA-1, GpsA-2, and GpsA-3) were prepared by coupling compound 2 with corresponding PT modified oligonucleotides. All the products were purified using Waters 2695 HPLC under the reversed-phase column (Agilent XBD-C18, 5µm, PN 990967-202) conditions: A, 0.05 mol/L triethylamine acetate (TEAA) solution (pH=7.0); B, acetonitrile; A: 90-60% in 30 min. The target product fractions were collected and characterized by electrospray ionization mass (ESI-MS, Sangon Biotech).

(b) Modification of fluorogenic oligonucleotide probes

The probe sequences 8nt-NH_2, 11nt-NH_2, 15nt-NH_2, and MM-NH_2 were conjugated with 7-azido-coumarin. All oligonucleotide probes were purified using a Waters 2695 HPLC under reversed-phase column (Agilent XBD-C18, 5μm, PN 990967-202). HPLC conditions: A, 0.05 mol/L triethylamine acetate (TEAA) solution (pH=7.0); B, acetonitrile; A: 90-65% in 25 min. The fractions containing the target products were collected and characterized using electrospray ionization mass spectrometry (ESI-MS, Sangon Biotech).

Table S1. List of modified oligonucleotide sequences in this study

Name	Sequence（5'-3'）	calculated	measured
GpsA-Ru-1	S—Ru(bpy)$_2$(phen)<br>G–P– AG AGA ACT GCG CTC GTA C<br>O	6501.0	6502.1
GpsA-Ru-2	S—Ru(bpy)$_2$(phen)<br>GAG–P– AGA ACT GCG CTC GTA C<br>O	6501.0	6502.0
GpsA-Ru-3	S—Ru(bpy)$_2$(phen)<br>GAG AG–P– A ACT GCG CTC GTA C<br>O	6501.0	6502.1
8nt-cou	GCG CAG TT-(7-AzC)	2933.2	2932.9
11nt-cou	CGA GCG CAG TT-(7-AzC)	3864.3	3865.0
15nt-cou	GTA CGA GCG CAG TTC-(7-AzC)	5103.2	5101.8
MM-cou	GCG CTG TT-(7-AzC)	2922.4	2923.8

4. The conversion rate.

This resulted in final concentrations for the target sequences of 250 nM, 100 nM, 50 nM, and 25 nM, respectively, while maintaining a final concentration of the probe sequence (8nt-cou) at 500 nM. The conversion rate was calculated by fitting the first-order dynamics curves depicted in Figure 2 with GraphPad's One-Phase Association model.

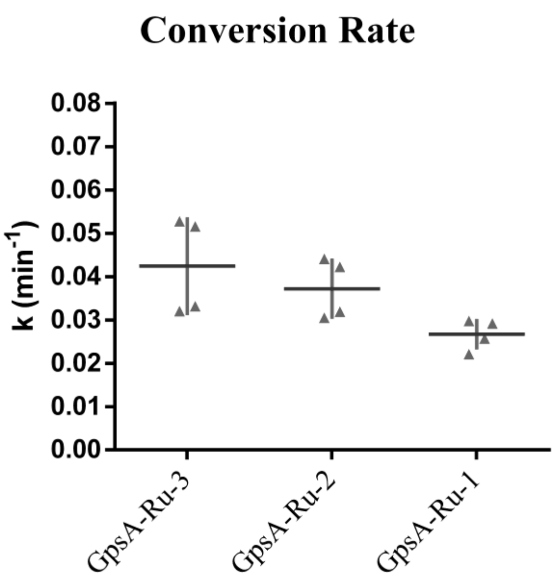

Figure S4 Conversion Rate

NMR and MS:

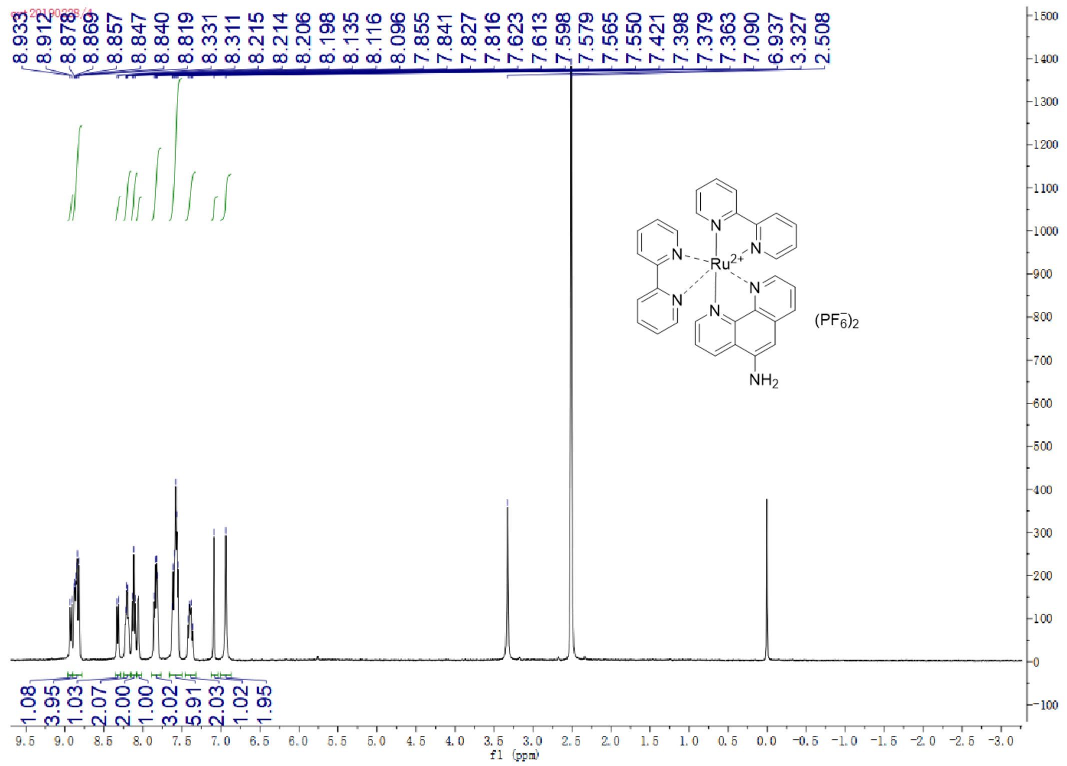

Figure S5 ^{1}H NMR of compound 1

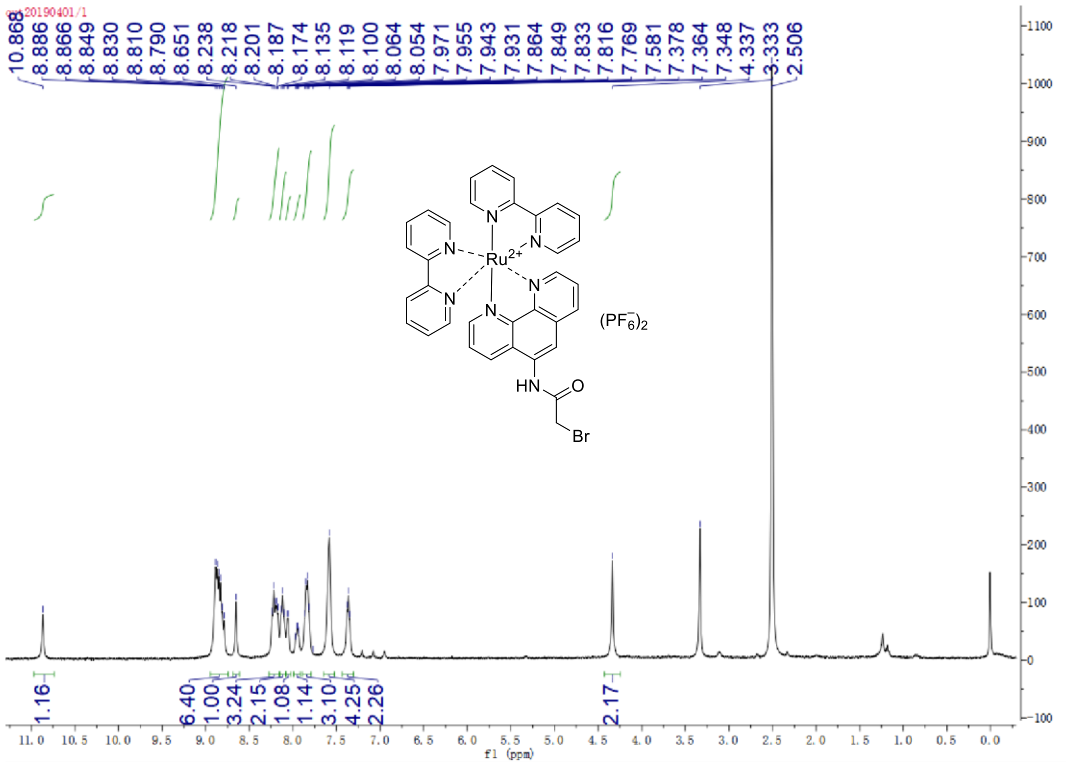

Figure S6 ^{1}H NMR of compound 2

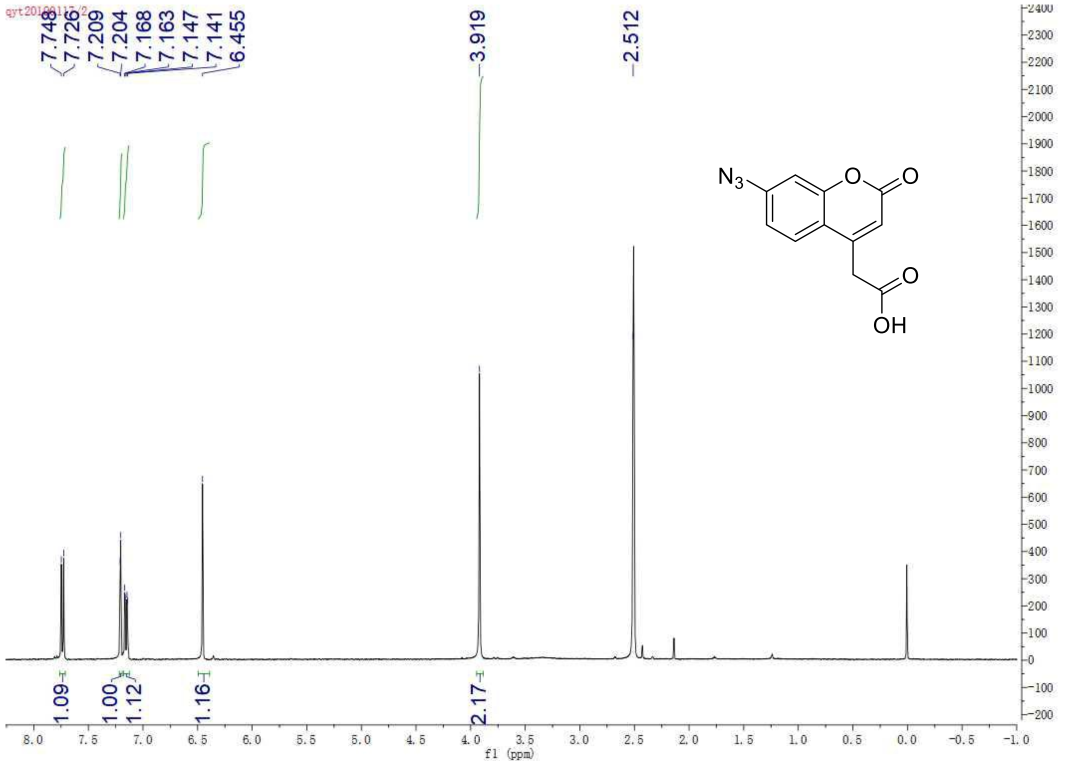

Figure S7 ^{1}H NMR of compound 3

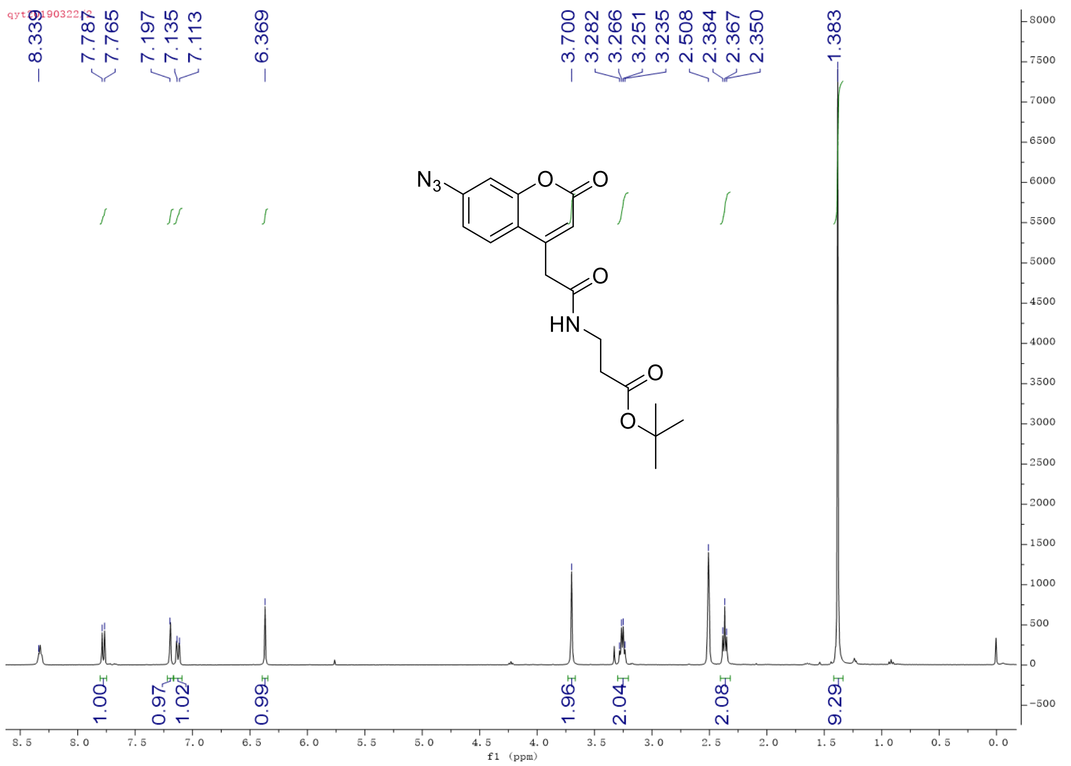

Figure S8 ^{1}H NMR of compound 4

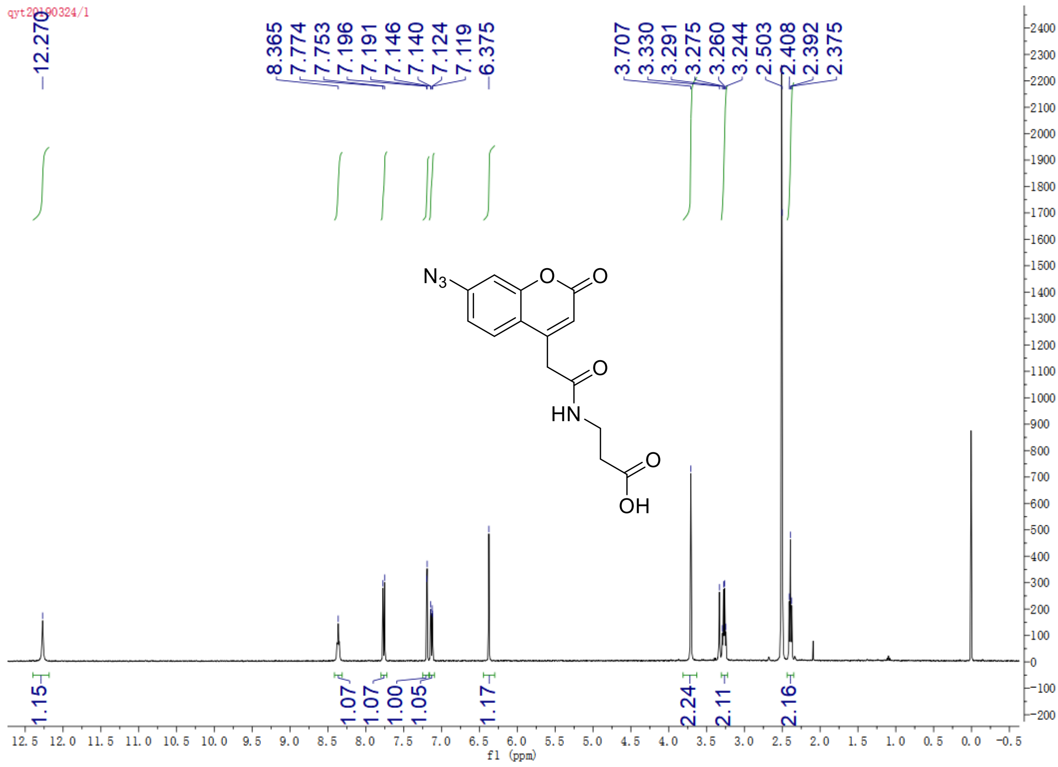

Figure S9 ^{1}H NMR of compound 5

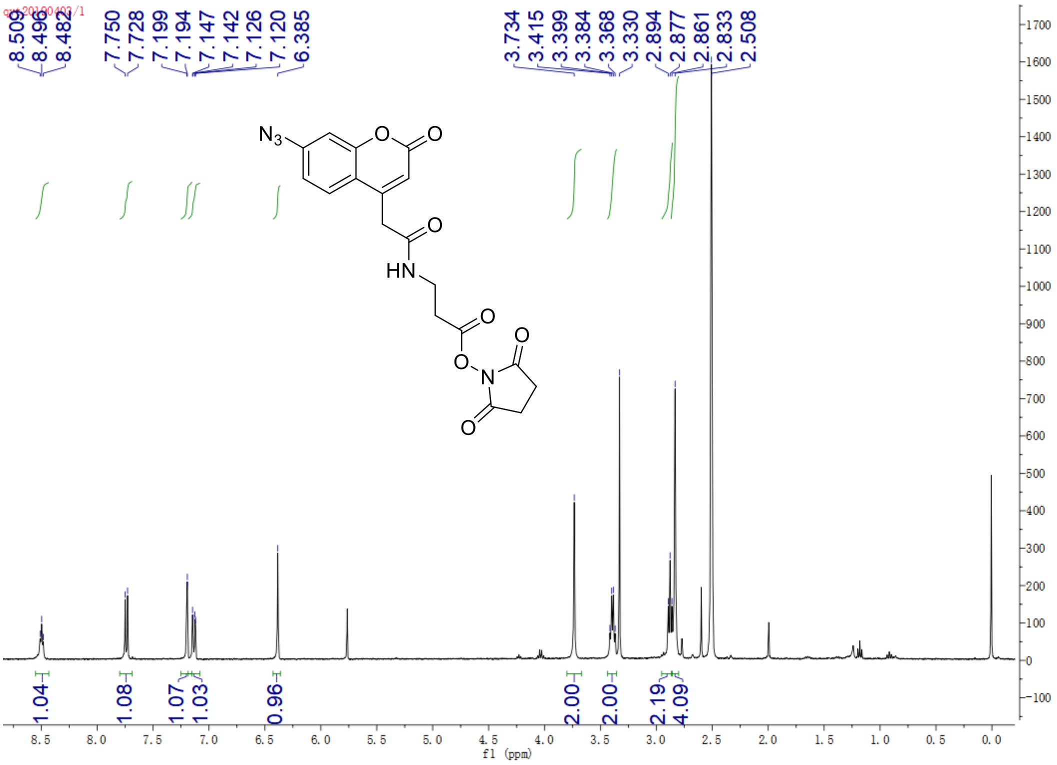

Figure S10 ^{1}H NMR of compound 6

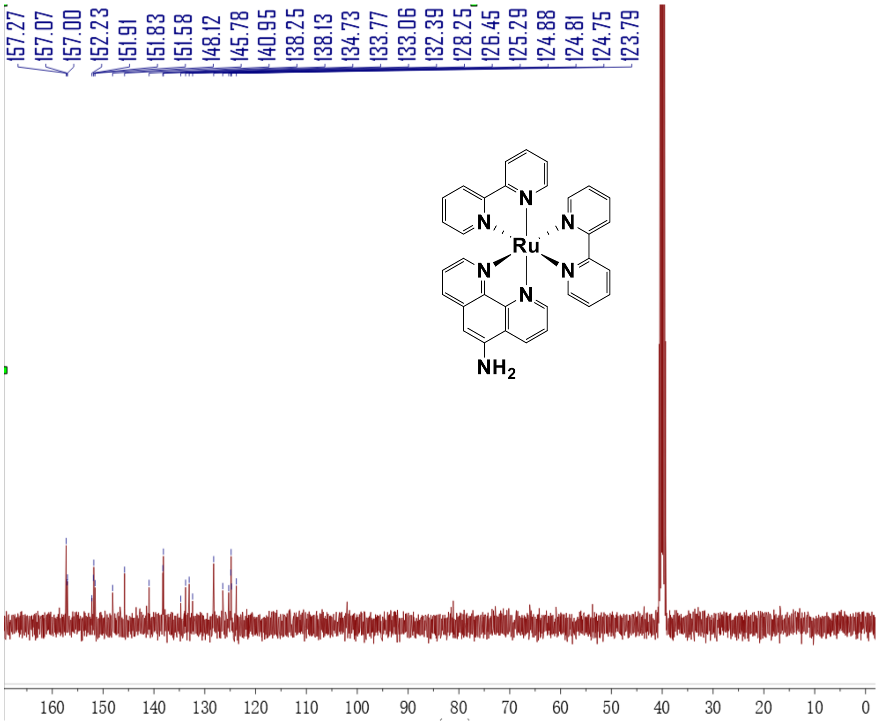

Figure S10 ^{13}C NMR of compound 1

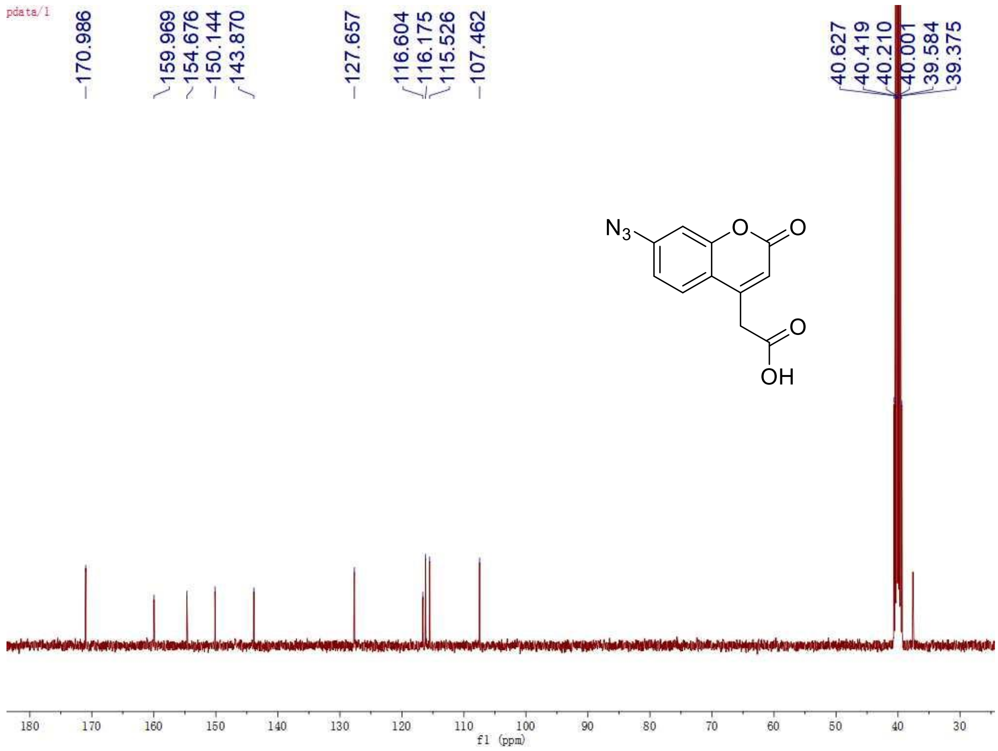

Figure S11 ^{13}C NMR of compound 3

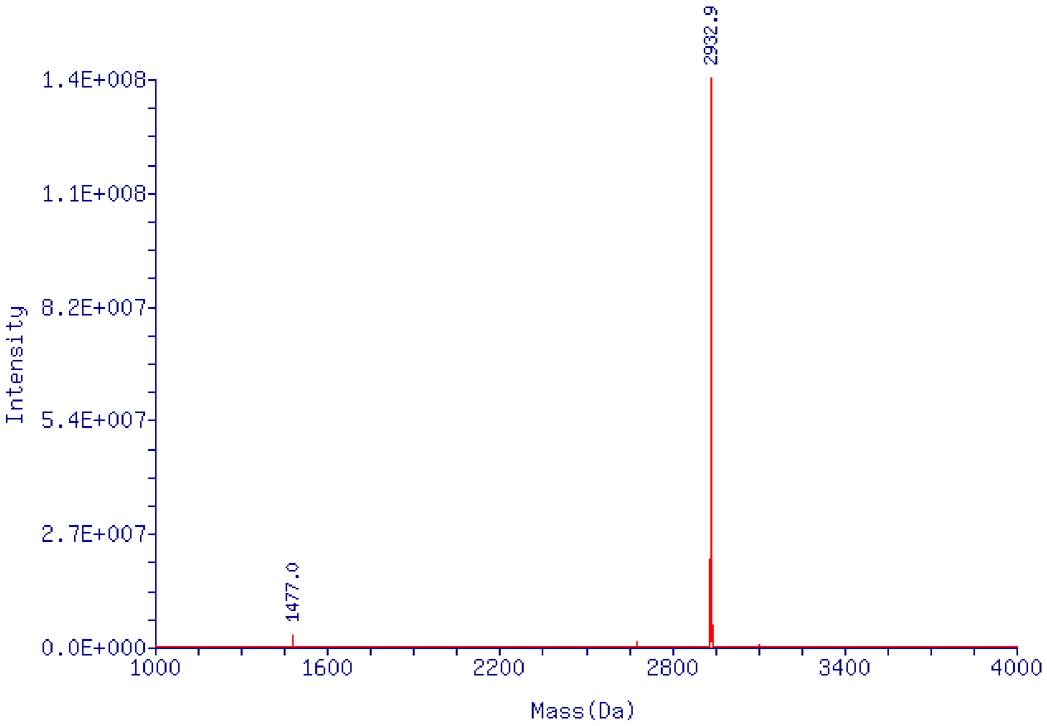

Figure S12 ESI-MS of 8nt-cou

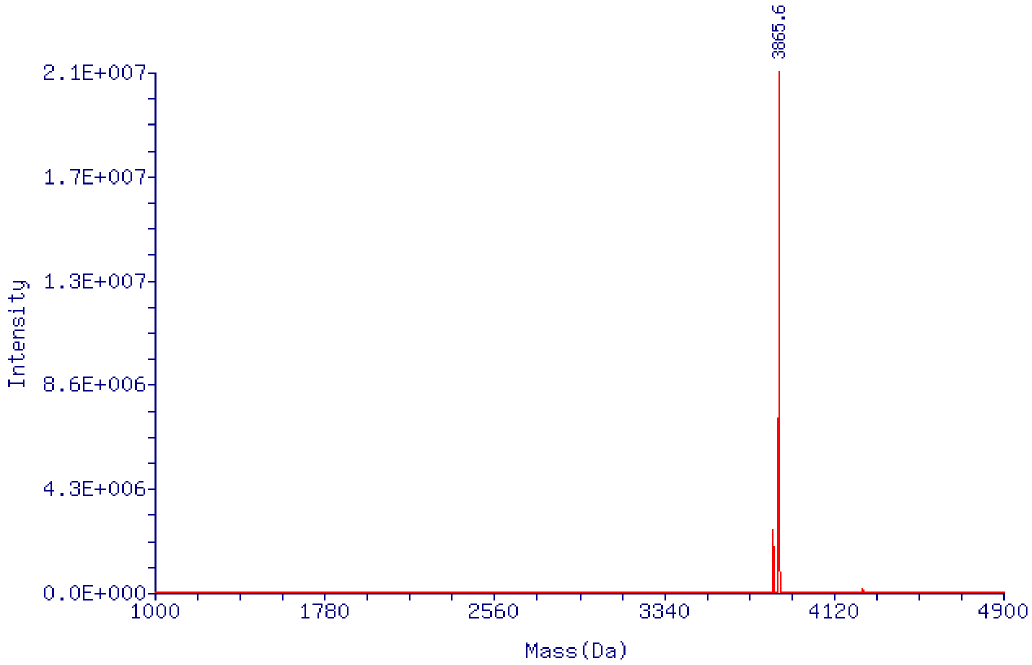

Figure S13 ESI-MS of 11nt-cou

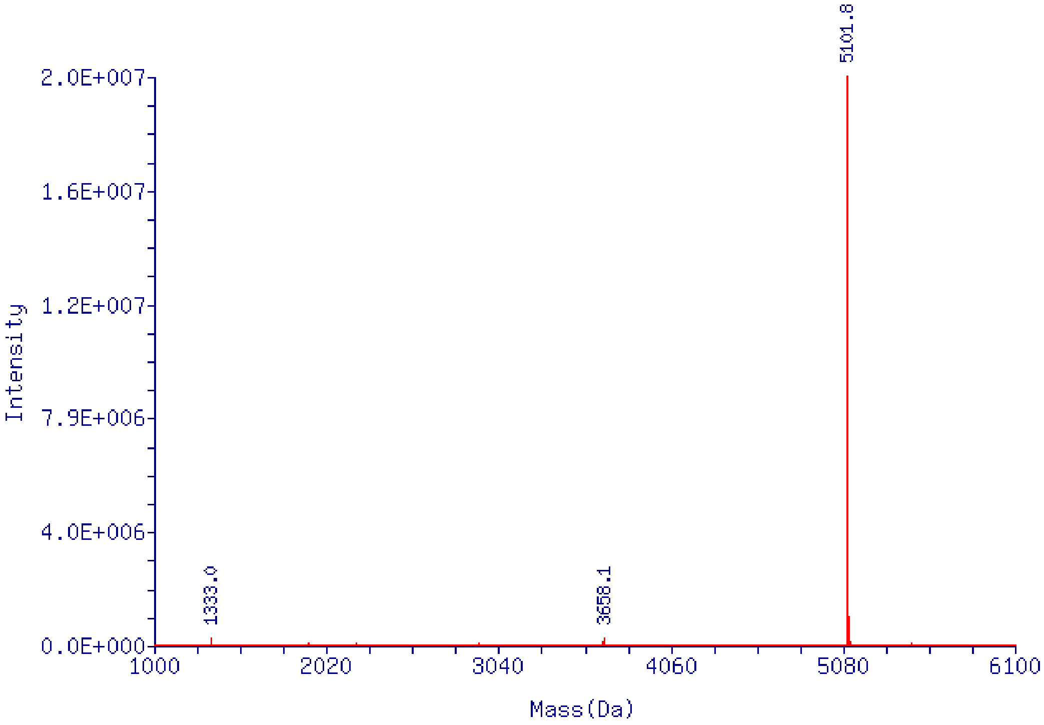

Figure S14 ESI-MS of 15nt-cou

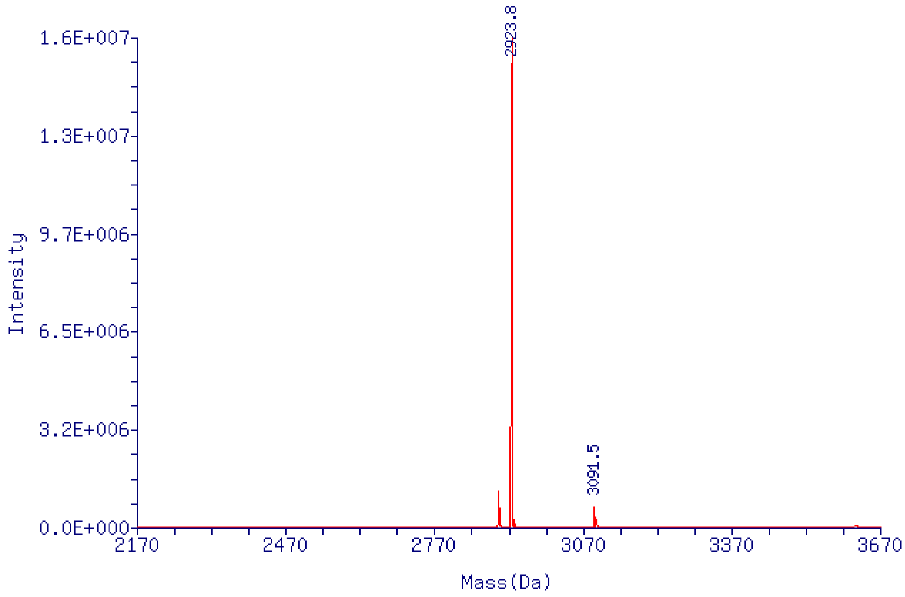

Figure S15 ESI-MS of MM-cou

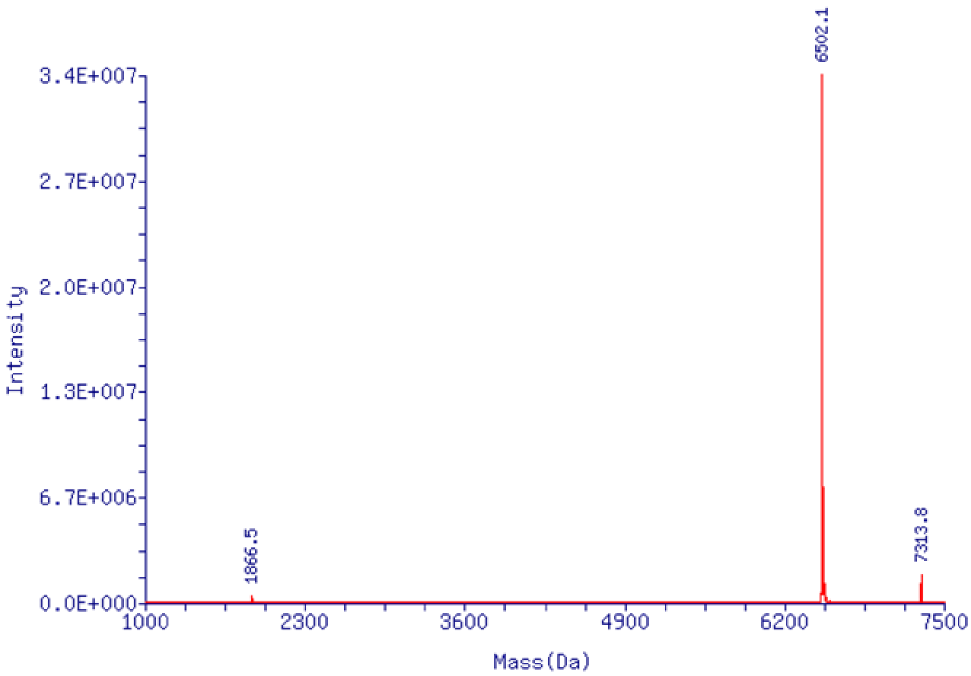

Figure S16 ESI-MS of GpsA-Ru-3
